# Prevalence of diabetes and pre-diabetes in rural Tehri Garhwal, India: influence of diagnostic method

**DOI:** 10.1186/s12889-019-7184-4

**Published:** 2019-06-24

**Authors:** Pam Anderson, Nathan Grills, Rajesh Singh, Rajkumari Singh, Roger G. Evans, Paramita Sengupta, Amanda G. Thrift

**Affiliations:** 1 Nossal Institute for Global Health, University of Melbourne, Melbourne, Australia; 2 Garhwal Community Development and Welfare Society, Mussoorie Road, Chamba, India; 3 Cardiovascular Disease Program, Biomedicine Discovery Institute and Department of Physiology, Monash University, Melbourne, Victoria Australia; 4 Christian Medical College, Ludhiana, India; 5 Department of Medicine, School of Clinical Sciences at Monash Health, Monash University, Clayton, Victoria 3800 Australia

**Keywords:** Anemia, Diabetes, Disadvantage, Fasting blood glucose, Glycosylated hemoglobin

## Abstract

**Background:**

There are few available data regarding the prevalence of diabetes in the sub-Himalayan region of India. The aim of this study was to determine the prevalence of pre-diabetes and diabetes in rural Garhwal based on glycosylated hemoglobin.

**Methods:**

In a cross-sectional survey of 500 adults from five randomly selected villages in Chamba, a mountainous Tehri Garhwal district in Uttarakhand in north-west India, we determined the prevalence of diabetes (hemoglobin (Hb) A_1c_ ≥ 6.5%) and pre-diabetes (5.7% ≤ HbA_1c_ ≤ 6.4%). In a sub-sample of those diagnosed with diabetes or pre-diabetes (*n* = 140), fasting blood glucose (FBG, *n* = 117) or postprandial blood glucose (PBG, *n* = 23), and blood hemoglobin concentration, was measured at follow-up.

**Results:**

Based on HbA_1c_, 10.0% had diabetes and 56.4% pre-diabetes. Of those diagnosed as diabetic by HbA_1c_, 10 of 16 (62.5%) were diagnosed as diabetic by FBG (> 125 mg/dL) or PBG (≥200 mg/dL). In those diagnosed as pre-diabetic by HbA_1c_, only 55 of 124 (44.4%) were diagnosed as pre-diabetic by FBG (100–125 mg/dL) or PBG (140–199 mg/dL). A large proportion of these 140 individuals (67.1%) were moderately to severely anemic (Hb < 11.4 mg/dL). The diagnostic gap for pre-diabetes between HbA_1c_ and FBG/PBG was similar for the groups with and without moderate to severe anemia.

**Conclusions:**

HbA_1c_ and FBG/PBG have similar diagnostic performance for diabetes in this population. However, many individuals were diagnosed with pre-diabetes by HbA_1c_ but not FBG/PBG. The relative excess diagnosis of pre-diabetes with HbA_1c_ does not appear to be explained by anemia, an endemic condition in India. The prognostic significance of diagnosis of pre-diabetes by HbA_1c_ but not FBG/PBG remains unknown, but merits investigation.

**Electronic supplementary material:**

The online version of this article (10.1186/s12889-019-7184-4) contains supplementary material, which is available to authorized users.

## Introduction

Worldwide, diabetes is a major contributor to morbidity and mortality [1]. In India the prevalence of diabetes and pre-diabetes is a growing public health concern. There are more than 69 million people with diabetes and this is estimated to increase to 80 million individuals by 2030 [2]. Despite the large number of people with diabetes there are few data about this condition in rural north India [3]. Most available data regarding the prevalence of diabetes in India come from populations in urban or southern regions [4–8]. But little is known about the mountainous regions of the north where poverty and poor access to health care facilities may exacerbate the problem.

Another factor that hampers studies of the prevalence of type 2 diabetes is the diagnostic methods available. The main diagnostic tests for diabetes include (1) fasting blood glucose; (2) random blood glucose; (3) glycosylated hemoglobin (HbA_1c_); and (4) the oral glucose tolerance test (OGTT). Each test has benefits and disadvantages. HbA_1c_ is now widely accepted for diagnosis [9, 10], providing a measure of glycemic control over the prior 3 months [11, 12], but is relatively expensive and has limited availability in some middle to low income countries [9]. While random blood glucose is more affordable and convenient as it requires no fasting, it is not recommended as a single diagnostic test unless classic symptoms of diabetes are present [9]. Fasting blood glucose and OGTT have been widely used as the standard diagnostic methods [13]. However, both tests have their limitations. The FBG test requires fasting, hence it can be problematic in an outpatient setting whereas OGTT is relatively expensive and has low reproducibility [13]. In reality, a mix of these screening tests are used in the diagnosis of diabetes in regions such as rural North India. It is unclear whether there are differences in the prevalence of diabetes in such settings according to the diagnostic test used.

HbA_1c_ is rapidly becoming the test of choice. The American Diabetes Association has recommended that HbA_1c_ may be used as a substitute to fasting blood glucose for diagnosing diabetes [9]. As the costs come down and the availability increases in India it is anticipated that these tests will become more widely used. Yet, in the context of North India, it is unknown how this test might perform. There is some evidence that anemia, and in particular consumptive anemias, may affect the HbA_1c_ result [14]. In particular, iron deficiency anemias may result in a spurious increase in HbA_1c_ [15], leading to a potential over-estimate of the prevalence of diabetes and pre-diabetes. Given that the proportion of people with anemia can approach 50% in this area [16], it is critical to determine whether anemia might affect HbA_1c_ in these mountainous regions in north-west India.

We aimed to determine the prevalence of pre-diabetes and diabetes in rural Garhwal based on glycosylated hemoglobin. Our primary hypothesis was that diabetes and pre-diabetes are highly prevalent in the region. Based on our initial finding of relatively high prevalence of both diabetes and pre-diabetes, we then aimed to test the hypothesis that HbA_1c_ over-diagnoses diabetes and pre-diabetes when compared to FBG. In addition, considering the presence of endemic anemia in India [17] and the possible confounding effect of anemia on HbA_1c_ as a diagnostic marker of diabetes [14], we also aimed to determine whether anemia is associated with over-diagnosis of pre-diabetes and diabetes.

## Methods

### Study design

We randomly selected five villages from 58 villages located on the west side of Chamba, in the mountainous Tehri District in the Garhwal region of the state of Uttarakhand in northern India (May to July 2015), using random number generation. We undertook a cross-sectional survey of adults in each village for between 8 and 10 days, with the aim of obtaining approximately 100 participants per village. As we did not have a census for each village, randomization of individual participants was not possible. Health staff, including a nurse and health workers, recruited participants following full informed consent. Ethical approval was obtained from the Garhwal Community Development and Welfare Society, and written informed consent was obtained before any data were collected from each participant.

#### Initial survey (time 1)

**Fig. 1:**
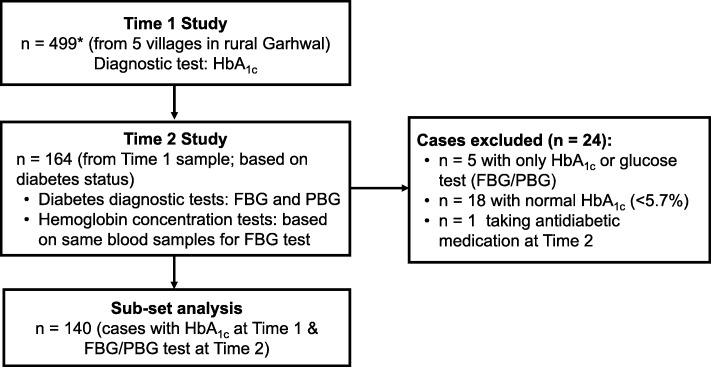
Study Flow Diagram. *HbA_1c_ at Time 1 was missing for 1 case, Average interval between Time 1 and Time 2 = 100 days, HbA_1c_: Glycosylated hemoglobin, FBG: Fasting blood glucose, PBG: Post-prandial blood glucose

We trained Accredited Social Health Activists (ASHAs), health-workers who reside in most villages in rural India, to administer a questionnaire and to conduct all the clinical and biochemical tests for this study. The ASHAs used questionnaire-based assessments to obtain details on age, sex, education, and monthly per-capita household income. ASHAs also measured height (Seca, Germany), weight (Salter, UK), and waist and hip circumference (Gulick, Patterson Medical, USA). Screening for diabetes and pre-diabetes was undertaken in 499 participants, using HbA_1c_ measured by the Afinion Analyzer (Alere, Oslo; Fig. 1). Staff from Chamba Hospital and a trained nurse were in the field to ensure clinical markers were properly measured.

#### Follow-up (time 2)

Approximately 3 months after the initial survey, a subset of individuals with diabetes or pre-diabetes were followed up with tests of fasting blood glucose and/or post-prandial blood glucose, and blood hemoglobin concentration (Fig. 1). As our aim was to provide management for people with diabetes and pre-diabetes, we aimed to follow-up only those with an HbA_1c_ ≥ 5.7% at the initial visit. The FBG test was conducted after fasting 8 to 10 h and the PBG was taken 2 h after a meal (75 g of glucose powder). Both FBG and PBG were assessed using an AccuSure glucometer (MicroGene, New Delhi). Using the same finger-prick blood samples for measuring FBG, blood hemoglobin concentration was assessed with a hemoglobinometer (Sahli’s type) by staff from Chamba Hospital.

### Clinical definitions

Diabetes and pre-diabetes were defined according to the American Diabetes Association’s cut points [9]. Participants with 5.7% ≤ HbA_1c_ ≤ 6.4% were classified as pre-diabetic and those with HbA_1c_ ≥ 6.5% were classified as diabetic. Pre-diabetes was also defined as 100 mg/dL ≤ FBG ≤ 125 mg/dL or 140 mg/dL ≤ PBG ≤ 200 mg/dL. Diabetes was defined as a FBG > 125 mg/dL or PBG > 200 mg/dL. Levels of HbA_1c_ and FBG were recoded into normal, pre-diabetes and diabetes based on these definitions. Moderate to severe anemia was defined as hemoglobin< 11.4 mg/dL adjusted for high altitude according to the World Health Organization’s criteria [18]. Hemoglobin concentration levels above the cut-off were classified as normal to mild anemia.

### Statistical analysis

All analyses were conducted using Stata version 14 (StataCorp., Texas). Age, weight, height, waist, hip, body mass index (BMI) and monthly income per capita (household monthly income/household size) are presented as medians and quartiles (Q1, Q3). Categorical variables (sex and education) are presented in percentages.

We assessed the crude prevalence of pre-diabetes and diabetes among the participants using HbA_1c_ measured during the initial survey (time 1). Age and gender specific prevalence of diabetes were compared using Pearson’s Chi-square test. Similarly, we compared age specific percentages of diabetes and pre-diabetes based on the FBG and/or PBG obtained in time 2 using Pearson’s Chi-square test.

The high prevalence of pre-diabetes at the initial survey prompted us to consider that anemia may have contributed to this surprising finding. To test whether HbA_1c_ over-diagnoses pre-diabetes and diabetes, we compared the proportions of the two conditions at time 1 with those determined by glucose tests taken at time 2 (August – October 2015) using McNemar’s Chi-square test. In the majority of cases, FBG was used (*n* = 117, 84%), but in those instances where this was unavailable (*n* = 23, 16%), PBG was used. We excluded one case who began treatment for diabetes between time 1 and time 2 (Fig. 1).

To further understand the influence of hemoglobin concentration on the level of HbA_1c_, we used McNemar’s test to compare the proportions of diabetes and pre-diabetes based on HbA_1c_ and glucose-based tests.

## Results

### Baseline characteristics

Of the 500 participants, 61% (303) were female and 39% (197) male. The median age of all participants was 50 years (IQR 42–60 years). More than half of the participants (61.4%) had no formal schooling or no more than 6 years of education. The median monthly income per capita was 1000 rupees (500–3000 rupees, *n* = 400 due to missing values for the number of people in the household). One person had missing information on HbA_1c_ (Fig. 1). There was a similar number of people recruited to each village (Additional file 1: Table S1).

There were notable differences in most of the anthropometric measures between individuals who were non-diabetic and those who were either pre-diabetic or diabetic according to HbA_1c_. Kruskal Wallis tests (set at 0.05 significance level) show that those with pre-diabetes or diabetes were progressively heavier, with greater BMI, waist and hip circumference, and waist-hip ratio (Additional file 2: Table S2).

### Prevalence of diabetes in rural Garhwal

Based on HbA_1c_, the overall prevalence of diabetes was 10% (95% CI 7.6–13%) and pre-diabetes 56.4% (95% CI 52–60.7%). Prevalence of pre-diabetes and diabetes was lowest in the youngest age group (Table 1).

**Table 1:** Diabetes status based on HbA_1c_ at Time 1, according to age group

Age group	Prevalence (95% CI)		
	Normal^a^	Pre-diabetes^b^	Diabetes^c^
0–44 years	44.3 (36.7 to 52.2)	48.7 (41.0 to 56.6)	7.0 (3.9 to 12.2)
45–59 years	29.8 (23.8 to 36.4)	58.0 (51.1 to 64.7)	12.2 (8.3 to 17.5)
≥60 years	27.2 (20.3 to 35.4)	63.2 (54.7 to 70.9)	9.6 (5.6 to 15.9)

Values represent percentages of cases within each age group with 95% confidence intervals in parentheses; *n* = 499; HbA_1c_ is missing for one case

^a^Normal was defined as HbA_1c_ < 5.7% (*n* = 168)

^b^Pre-diabetes was defined as 5.7% ≤ HbA1c ≤ 6.4% (*n* = 282)

^c^Diabetes was defined as HbA1c ≥6.5% (*n* = 49)

There was a strong association between diabetes status and age (χ^2^ = 13.3; *p* = 0.01), but we were unable to detect a statistically significant association between gender and prevalence of pre-diabetes or diabetes (χ^2^ = 1.07; *p* = 0.59).

### Glycosylated hemoglobin vs fasting blood glucose/post-prandial blood glucose

Among the 140 people we followed-up, we could detect no significant change in anthropometric variables between the times of measurement of HbA_1c_ and FBG/PBG (Table 2). Of these 140 people, 124 (88.6%) were pre-diabetic according to HbA_1c_ cut-offs compared to 56 (40.0%) using FBG/PBG (Table 3). This corresponds to a 48.6% greater percentage of pre-diabetes using HbA_1c_ than by FBG/PBG (95% CI: 39.3 to 57.7%) (McNemar’s chi-square = 66.1; *p* < 0.001).

**Table 2:** Age, anthropometric and biochemical characteristics of participants with HbA_1c_ and glucose-based test

	Time 1^a^	Time 2^a^	*p*-value
Age, years	52 (18)	NA	NA
HbA_1c_ (%)	5.9 (5.8–6.2)	NA	NA
Height, cm	154.5 (149.5–161.3)^b^	As for Time 1	NA
Weight, kg	56.0 (48.8–64.0)^b^	55.0 (46.6–63.4)	0.06
Body mass index, kg/m^2^	22.7 (19.8–25.7)^c^	22.3 (19.6–25.7)^b^	0.08
Waist, cm	80.7 (70.0–89.6)	81.3 (71.5–89.8)	0.22
Hip, cm	93.0 (88.2–97.8)	92.8 (88.0–97.5)	0.24
Waist-hip ratio	0.87 (0.8–0.9)	0.88 (0.8–0.9)	0.79
FBG, mg/dL	NA	104 (95–114)^d^	NA
PBG, mg/dL	NA	130 (111–152)^e^	NA
Hemoglobin, g/dL	NA	11 (10–12)	NA

Data show median of baseline characteristics with interquartile range in parentheses

*n* = 140, excluding 18 subjects with normal HbA_1c_ results (HbA_1c_ < 5.7%)

*p*-values were based on Wilcoxon’s signed-rank test

^a^Diagnostic test for diabetes at Time 1: HbA_1c_; at Time 2: FBG/PBG test

^b^Time 1 Height & Weight; Time 2 BMI: *n* = 139

^c^Time 1 BMI: *n* = 138

^d^Time 2 FBG: *n* = 117

^e^ Time 2 PBG: *n* = 135

*BMI* body mass index, *HbA*_*1c*_ glycosylated hemoglobin, *FBG* fasting blood glucose, *PBG* post-prandial blood glucose, *NA* not applicable/available

**Table 3:** Diabetic status based on fasting blood glucose or post-prandial glucose, among those diagnosed as pre-diabetic or diabetic using HbA_1c_

Diagnosis based on FBG/PBG^b^	Diagnosis based on HbA_1c_^a^		
	Pre-diabetes	Diabetes	Total
No Diabetes	61 (49.2%)	5 (31.2%)	66
Pre-diabetes	55 (44.4%)	1 (6.3%)	56
Diabetes	8 (6.5%)	10 (62.5%)	18
Total	124 (100%)	16 (100%)	140

Values are number of cases and column percentages

*HbA*_*1c*_ glycosylated hemoglobin, *FBG* fasting blood glucose, *PBG* post-prandial blood glucose

^a^Tested at time 1; pre-diabetes was defined as 5.7% ≤ HbA_1c_ ≤ 6.4%; diabetes was defined as HbA_1c_ ≥ 6.5%

^b^Tested at time 2; pre-diabetes was defined as 100 mg/dL ≤ FBG ≤ 125 mg/dL or 140 mg/dL ≤ PBG ≤ 200 mg/dL; diabetes was defined as FBG > 125 mg/dL or PBG > 200 mg/dL

In the instances where FBG was not available, diagnosis was based on PBG

There was no evidence that diabetes was over-diagnosed when HbA_1c_ was used as the diagnostic criterion. The difference in proportions of diabetes assessed by HbA_1c_ and FBG/PBG was − 0.02 (95% CI: − 0.07 to 0.05; McNemar’s χ^2^ = 0.29; *p* = 0.79).

### Hemoglobin, HbA_1c_ and fasting blood glucose

In both people with and without moderate to severe anemia, a much smaller proportion were diagnosed with pre-diabetes by FBG/PBG than HbA_1c_ (Table 4). The difference between the two diagnostic criteria was consistent across the two categories of hemoglobin concentration [no anemia or mild anemia: 0.48 (0.37–0.61); McNemar’s χ^2^ = 44.1; *p* < 0.01; moderate to severe anemia: 0.48 (0.31–0.64), McNemar’s χ^2^ = 22; *p* < 0.01]. Similarly, the proportions categorized by HbA_1c_ and FBG/PBG as diabetic did not differ markedly according to category of blood hemoglobin concentration (Table 4).

**Table 4:** Diabetic status based on glycosylated hemoglobin and glucose-based tests, according to anemia status

	No/Mild Anemia^a^ *n* = 46		Moderate/Severe Anemia^a^ *n* = 94	
	HbA_1c_^b^	FBG/PBG^c^	HbA_1c_^b^	FBG/PBG^c^
No Diabetes	–	21 (45.7%)	–	45 (47.9%)
Pre-diabetes	39 (84.8%)	17 (37.0%)	85 (90.4%)	39 (41.5%)
Diabetes	7 (15.2%)	8 (17.4%)	9 (9.6%)	10 (10.6%)

Only cases with both HbA_1c_ at Time 1 and FBG/PBG at Time 2 were included in analysis; *n* = 140, excluding 18 subjects with normal HbA_1c_ results (HbA_1c_ < 5.7%)

^a^No/mild anemia was defined as Hb > 11.4 g/dL; moderate/severe anemia was defined as Hb ≤ 11.4 g/dL

^b^Pre-diabetes was defined as 5.7% ≤ HbA1c ≤ 6.4%; diabetes was defined as HbA1c ≥6.5%

^c^Pre-diabetes was defined as 100 mg/dL ≤ FBG ≤ 125 mg/dL or 140 mg/dL ≤ PBG ≤ 200 mg/dL; diabetes was defined as FBG > 125 mg/dL or PBG > 200 mg/dL

In the instances where FBG was not available, diagnosis was based on PBG (*n* = 23)

## Discussion

The prevalence of both pre-diabetes and diabetes in our study sample was generally much higher than other states of India and other South Asian countries [4, 19]. We observed a large disparity between proportions of the sample diagnosed with pre-diabetes based on HbA_1c_ and FBG/PBG. However, we were unable to detect a difference in the proportions of diabetics diagnosed by the two tests. It has been suggested that anemia can influence HbA_1c_ levels, and thus the performance of this diagnostic test [14]. However, our data indicate that the diagnostic gap between pre-diabetes based on HbA_1c_ and FBG/PBG is unlikely to be attributable to confounding by the high prevalence of anemia in the population we studied.

### Proportions of diabetes and pre-diabetes

Our findings indicate that HbA_1c_ has comparable performance to FBG/PBG with regard to diagnosis of diabetes in this rural population. HbA_1c_ and glucose tests both detected very similar proportions of diabetes in the sample we studied. Our finding is consistent with those of two recent studies, one in an out-patient clinic in China and another in a tertiary care setting in India [20, 21]. However, substantial disparities between diagnoses based on HbA_1c_ and glucose-based tests have previously been reported [4, 22–26]. In some cases HbA_1c_ diagnosed much larger proportions of diabetes than did FBG [4, 23–25] while in other studies HbA_1c_ diagnosed smaller percentages of diabetes than did FBG [22, 26]. These studies varied in settings ranging from general communities as well as sea-level and high-altitude settings, covering various ethnic groups including Arabs, Asian-Americans, Native Hawaiians and Peruvians. Given that there is evidence of HbA_1c_ levels being associated with both genetic factors [11, 27, 28] and geographical settings [23] further investigation of HbA_1c_-FBG relationship in rural mountainous regions are required.

In contrast to the diagnosis of diabetes, 49% of study subjects who were diagnosed with pre-diabetes based on HbA_1c_ were classified as normal by measurement of FBG/PBG. Our findings are consistent with those of two recent studies which indicate considerable proportions of individuals who are pre-diabetic based on HbA_1c_ criteria have normal FBG results [24, 25]. However, others found that smaller proportions of pre-diabetics were identified by HbA_1c_ than glucose-based methods [26, 29]. The divergence is unlikely to reflect device measurement errors, given the evidence that the point-of-care device used in our study has comparable performance to a laboratory based HbA_1c_ test [30, 31]. Despite the fact that FBG and OGTT have both been widely used as the gold standards for diagnosing diabetes, there is still no uniform definition of pre-diabetes [32–35]. Marked discordance between pre-diabetes by HbA_1c_ and FBG/PBG criteria in the present study therefore does not necessarily imply false positives. In fact, there is some evidence that HbA_1c_ and glucose-based diagnoses identify different populations within the hyperglycemic category [27, 29]. Both tests may offer uniquely important prognostic information.

The presence of a large proportion of individuals diagnosed as having pre-diabetes based on HbA_1c_ but with normal glucose-based results may have important prognostic implications. HbA_1c_’s ability to predict major clinical complications such as cardiovascular disease is of major prognostic significance [36–38]. For example, HbA_1c_ levels were able to predict lipid profile, a key determinant of cardiovascular heart disease [37, 38]. Indeed, in a community-based study of non-diabetic, middle-aged adults in four U.S. communities, Selvin and colleagues observed that elevated HbA_1c_ (≥6%) was strongly associated with the risks of cardiovascular disease, all-cause mortality and ischemic stroke [38]. The associations remained strong and significant after adjusting for baseline FBG levels. Moreover, in individuals with stable coronary artery disease, HbA_1c_ values of 6.3% or greater were linked with adverse cardiovascular outcomes [36]. There is also evidence that reducing HbA_1c_ by 0.2% can lead to a 10% reduction in risk of mortality within 12 months [37]. Whether the increased risk of cardiovascular diseases associated with elevated HbA_1c_ is due to pre-diabetic conditions or the subsequent development of diabetes remains unclear [39, 40]. However, the additional clinical information provided by a measurement of HbA_1c_ in the pre-diabetic range may render HbA_1c_ a more cost-effective option than FBG or OGTT, particularly in areas where comprehensive medical tests are less accessible and populations are more predisposed to cardiovascular diseases. Further research on this subject should be pursued in order that this additional prognostic benefit can be fully realized.

Another possible prognostic implication of pre-diabetes diagnosed by HbA_1c_ but not FBG/PBG lies in the prediction of progression to diabetes. In a systematic review of studies investigating the performance of HbA_1c_ in predicting progression to diabetes among adults aged 18 years and over, Zhang and colleagues observed that HbA_1c_ ≥ 6.0% was associated with a very high risk of subsequent development of diabetes [41]. Pre-diabetes identified by fasting glucose and oral glucose tolerance tests, on the contrary, had limited ability to predict progression to diabetes [42]. On the other hand, others found similar rates of progression to diabetes in those identified as pre-diabetic by a single FPG test compared to a HbA_1c_ test [43, 44]. Regardless, our current findings indicate that the prevalence of pre-diabetes in rural Garhwal is much higher than the national rural prevalence [4]. Given that timely lifestyle changes can be effective in preventing or delaying progression to diabetes [33], the advantages of using HbA_1c_ for mass screening of pre-diabetes in low-income rural areas may outweigh its disadvantages in the long run.

### Association with hemoglobin

Consumptive anemia can confound the relationship between HbA_1c_ level and glycemic control [14, 45]. Thus, it could potentially account for the observed difference in diagnoses of pre-diabetes by HbA_1c_ and FBG. However, we found that both the pattern and magnitude of differences in the proportions of pre-diabetes and diabetes diagnosed by HbA_1c_ compared with FBG/PBG, were independent of anemia status. There is prior evidence of substantial changes in HbA_1c_ levels in the presence of anemia [14, 15]. In a recent systematic review of the effects of anemia and abnormalities of erythrocyte indices on HbA_1c_, the authors suggested that iron deficiency, and particularly iron deficiency anemia, may lead to an increase in HbA_1c_ [14]. In addition, relatively larger divergence was found between diagnoses of pre-diabetes and diabetes based on HbA_1c_ and OGTT, in a subgroup of young anemic adults who were deficient in iron, B12 and folic acid, when compared to a reference group [15]. The association between HbA_1c_ and hemoglobin concentration was also found to vary according to the forms of anemia. While iron deficiency anemia was associated with elevated HbA_1c_ [15], some forms of anemia were found to be linked with diminished HbA_1c_ level. A typical example is hemolytic anemia, in which the lifespan of erythrocytes is shortened, causing a drop in HbA_1c_ [14]. Other conditions which may decrease levels of HbA_1c_ include acute hemorrhage and hemoglobinopathies [46]. However, so far there is no consistent evidence regarding the influence of anemia and hemolytic disorders on HbA_1c_.

### Limitations

Because of the difference in timing between HbA_1c_ and FBG tests, caution should be applied in the interpretation of these data. Participants were told their level of HbA_1c_ immediately after the test. Thus, some of the disparity between diagnosis of pre-diabetes by HbA_1c_ and FBG/PBG may be due to altered behavior between the two time points. However, lifestyle changes, if any, were unlikely to have contributed to the large disparity, given the minor differences in weight, body mass index and waist-hip ratio between time 1 and time 2. Furthermore, because we did not follow-up people who were categorized as having a normal HbA_1c_ at baseline, we will have missed detecting anyone who was negative for diabetes and pre-diabetes at the first assessment, but who were then positive using the glucose-based definition. This may mean that the differences that we observed are overestimated. We also acknowledge that our conclusions may not be generalizable across the whole region of Tehri-Garhwal because only five villages were surveyed. However, our findings provide preliminary evidence for a particularly high prevalence of diabetes and poor glycemic control in the sub-Himalayan region. They therefore provide the impetus for larger studies, including sampling of participants in a manner that represents the wider population of the region, to fully characterize the prevalence of diabetes in mountainous areas of North India.

## Conclusions

Our study is one of the few in which HbA_1c_ has been utilized to diagnose diabetes in the mountainous rural communities in North India. The remarkably high prevalence of diabetes and pre-diabetes indicates the need for more in-depth research, including identification of the underlying determinants of diabetes and pre-diabetes in this region. More region-specific studies are needed in North India, especially where poverty and ethnic diversity are more pronounced. In view of the marked disparity in diagnosis of pre-diabetes between HbA_1c_ and FBG, health-care professionals should be well-informed of the complex determinants of HbA_1c_ to maximize the benefits of the HbA_1c_ test. From a policy perspective, there is a pressing need for advocacy of effective, tailor-made public health initiatives to prevent and respond to this imminent diabetes crisis in the rural mountainous regions of North India.

## Additional files

Additional file 1 **Table S1.** Distribution of participants according to village. (DOCX 14 kb)

Additional file 2 **Table S2.** Age, anthropometric and biochemical characteristics of participants at baseline, according to diabetes status as measured by HbA_1c_. (DOCX 18 kb)

## Data Availability

The datasets generated and/or analysed during the current study are not publicly available because these are small communities and publication of such data would compromise anonymity. However, a limited dataset, without age and other potentially identifying information, is available from the corresponding author on reasonable request.

## Abbreviations

ASHAs: Accredited Social Health Activists
BMI: Body mass index
FBG: Fasting blood glucose
HbA_1c_: Glycosylated hemoglobin
OGTT: Oral glucose tolerance test
PBG: Post-prandial blood glucose
