# Supplementary material for: Prevalence of diabetes and pre-diabetes in rural Tehri Garhwal, India: influence of diagnostic method
Source: BMC Public Health. 2019 Jun 24;19:817. doi: 10.1186/s12889-019-7184-4 (PMC6591826; doi:10.1186/s12889-019-7184-4)
Supplement: Supplementary file 1 — Table S1. Distribution of participants according to village. (DOCX 14 kb) [file 12889_2019_7184_MOESM1_ESM.docx]

**Table S1. Distribution of participants according to village**

| Village | Measured HbA_1c_ at Time 1  n = 499 (%) | Measured FBG at Time 2  n = 117 (%) | Measured PBG at Time 2  n = 23 (%) |
| --- | --- | --- | --- |
| Chodiyalgaon | 100 (20.0) | 25 (21.4) | 13 (56.5) |
| Jaripani | 100 (20.0) | 23 (19.7) | 1 (4.4) |
| Pursolgaon | 104 (20.8) | 15 (12.8) | 1 (4.4) |
| Sangaon | 99 (19.8) | 26 (22.2) | 0 (0.0) |
| Silkotee | 96 (19.2) | 28 (23.9) | 8 (34.8) |
