# Supplementary material for: Prevalence of diabetes and pre-diabetes in rural Tehri Garhwal, India: influence of diagnostic method
Source: BMC Public Health. 2019 Jun 24;19:817. doi: 10.1186/s12889-019-7184-4 (PMC6591826; doi:10.1186/s12889-019-7184-4)
Supplement: Supplementary file 2 — Table S2. Age, anthropometric and biochemical characteristics of participants at baseline, according to diabetes status as measured by HbA1c. (DOCX 18 kb) [file 12889_2019_7184_MOESM2_ESM.docx]

**Table S2.** Age, anthropometric and biochemical characteristics of participants at baseline, according to diabetes status as measured by HbA_1c_.

| Characteristics | Normal  n = 168 | Pre-diabetes  n = 282 | Diabetes  n = 49 | p-value^b^ |
| --- | --- | --- | --- | --- |
| Age, years | 47 (40-57.5) | 52 (42-61) | 52 (46-60) | <0.01 |
| Female | 107 (63.7)^a^ | 166 (58.9)^a^ | 29 (59.2)^a^ | 0.59^c^ |
| HbA_1c_, % | 5.5 (5.3-5.6) | 5.9 (5.8-6.1) | 6.9 (6.5-7.8) | NA |
| Height, cm | 154.9 (148.6-161) | 155.5 (149.5-162) | 155.5 (151.4-163) | 0.32 |
| Weight, kg | 51.4 (45-58.3) | 55 (48.8-63.1) | 57.4 (51.5-64.2) | <0.01 |
| Body mass index, kg/m^2^ | 21.1 (18.9-23.8) | 22.6 (19.9-25.4) | 23.7 (21.2-25.4) | <0.01 |
| Waist, cm | 72.2 (61-82.6) | 80 (70-88) | 84.05 (75.5-91.7) | <0.01 |
| Hip, cm | 89 (82.2-95.2) | 92 (87.4-96.5) | 95.6 (89-99) | <0.01 |
| Waist-hip ratio | 0.86 (071-0.92) | 0.87 (0.78-0.93) | 0.89 (0.83-0.93) | 0.02 |

Data show median of baseline characteristics with interquartile range in parentheses unless otherwise indicated.

Normal was defined as HbA_1c_< 5.7%; Pre-diabetes was defined as 5.7% ≤ HbA1c ≤ 6.4%; Diabetes was defined as HbA1c ≥6.5.

^a^ n (%)

^b^ p-values were based on Kruskal-Wallis test (baseline characteristics and diabetes status as measured by HbA_1c_).

^c^ p-value was based on Pearson’s Chi-square test (age and diabetes status as measured by HbA_1c_).
